# FLT3 inhibitors as maintenance therapy post allogeneic hematopoietic stem cell transplantation in acute myeloid leukemia patients with FLT3 mutations: A meta‐analysis

**DOI:** 10.1002/cam4.5480

**Published:** 2022-11-21

**Authors:** Xinhong Fei, Shuqin Zhang, Jiangying Gu, Jingbo Wang

**Affiliations:** ^1^ Department of Hematology Aerospace Center Hospital Beijing China

**Keywords:** acute myeloid leukemia, allogeneic hematopoietic stem cell transplantation, Fms‐like tyrosine kinase 3 inhibitor, maintenance therapy

## Abstract

**Background:**

Acute myeloid leukemia (AML) patients with a Fms‐like tyrosine kinase 3 (FLT3) mutation have a high incidence of relapse despite allogeneic hematopoietic stem cell transplantation (allo‐HSCT) and a subsequent poor prognosis. FLT3 inhibitors (FLT3i) have been suggested to reduce the post‐transplant relapse risk in recent studies. As more evidence is accumulated, we perform the present meta‐analysis to assess the efficacy and safety of FLT3i as post‐transplant maintenance therapy in AML patients.

**Methods:**

Literature search was performed in public databases from inception to December 31, 2021. Overall survival (OS), relapse‐free survival (RFS), cumulative incidence of relapse (CIR), non‐relapse mortality (NRM), graft‐versus‐host disease (GVHD) and adverse events were compared between FLT3i and control groups. Pooled hazard ratio (HR) or relative risk (RR) with corresponding 95% confidence interval (CI) were calculated.

**Results:**

We identified 12 eligible studies with 2282 FLT3‐mutated AML patients who had received HSCT. There was no between‐study heterogeneity and a fix‐effect model was used. Post‐transplant FLT3i maintenance significantly prolonged OS (HR = 0.41, 95%CI: 0.32–0.52, *p* < 0.001) and RFS (HR = 0.39, 95%CI 0.31–0.50, *p* < 0.001), and reduced CIR (HR = 0.31, 95%CI 0.20–0.46, *p* < 0.001) as compared with control. There were no significant risk differences in NRM (RR = 0.69, 95%CI 0.41–1.17, *p* = 0.169), acute GVHD (RR = 1.17, 95%CI 0.93–1.47, *p* = 0.175), chronic GVHD (RR = 1.31, 95%CI 0.91–1.39, *p* = 0.276) and grade ≥3 adverse events between both groups, except for skin toxicity (RR = 5.86, 95%CI 1.34–25.57, *p* = 0.019).

**Conclusion:**

Post‐transplant FLT3i maintenance can improve survival and reduce relapse in FLT3‐mutated AML patients and is tolerable.

## Graphical abstract

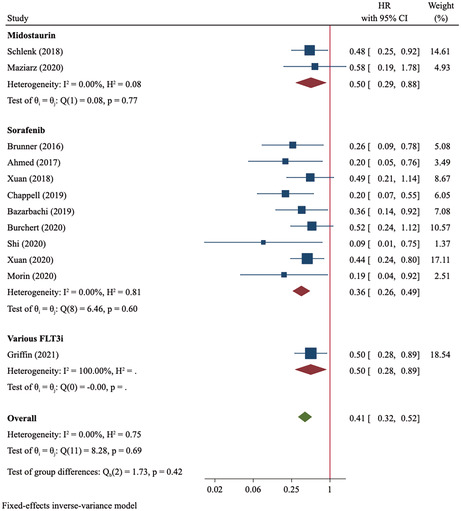

## 1 INTRODUCTION

Acute myeloid leukemia (AML) is a heterogenous hematological malignancy and represents the most common type of acute leukemia in adults worldwide.^1^ It derives from clonal myeloid stem cells with a series of cytogenetic abnormalities and molecular mutations and is characterized by the accumulation of myeloid progenitor cells.^2^ The overall prognosis of AML is largely determined by the genomic profiles.^3^

Fms‐like tyrosine kinase 3 (FLT3) mutations are frequently found in AML patients. The most common FLT3 mutations, that is, internal tandem duplication (ITD), are within the juxtamembrane domain and occur in 20 ~ 30% of newly diagnosed patients.^4^ Point mutations in tyrosine kinase domain (TKD) of FLT3 are less common and detected in about 7% of patients.^5^ These mutations constitutively activate FLT3 receptors and then dysregulate multiple downstream pathways, including phosphatidylinositol 3‐kinase (PI3K), rat sarcoma (Ras) and the signal transducers and activators of transcription 5 (STAT5) signaling.^6^, ^7^, ^8^ Subsequently, the aberrant signaling transductions promote proliferation, impair differentiation and resist apoptosis of leukemic cells.^9^, ^10^ The presence of FLT3‐ITD mutations is strongly associated with elevated relapse risk and a worsened prognosis.^11^, ^12^ Yet, the prognosis impact of TLT3‐TKD mutations is still uncertain.^13^

Since FLT3 mutations portend a poor prognosis, allogeneic hematopoietic stem cell transplantation (allo‐HSCT) is recommended as standard of care for this group of patients to improve survival.^14^, ^15^ However, post‐transplant outcomes are still highly dependent on FLT3 mutational status, and the relapse rate remains high which is a major reason of patient death despite HSCT.^16^, ^17^ After allo‐HSCT, patients with FLT3‐ITD mutations have relapse incidences at 2‐ or 3‐year follow‐ups as nearly twice as those without mutations, which translate to a significantly shorter overall survival (OS).^18^, ^19^ Once relapsing after transplantation, patients rarely have effective treatment options since second HSCT, chemotherapy, FLT3i and donor lymphocyte infusion (DLI) only achieve long‐term outcomes in a small proportion of relapsed patients.^20^, ^21^, ^22^ This highlights the importance of post‐HSCT maintenance therapy for the prevention of relapse in FLT3‐mutated patients.

FLT3 inhibitors (FLT3i) are a group of tyrosine kinase inhibitors that target signaling pathways triggered by FLT3 mutations. Various FLT3i are currently explored for clinical application at different treatment stages of FLT3‐mutated AML patients, including induction, maintenance pre‐ and post‐HSCT, and salvage therapy.^23^ Recent observational studies and clinical trials demonstrated that post‐transplant maintenance therapy using FLT3i could reduce relapse risk and improve survival in FLT3‐mutated AML patients.^24^, ^25^, ^26^ With growing evidence, we performed the present meta‐analysis to evaluate the efficacy and safety of FLT3i as maintenance therapy following allo‐HSCT in AML patients with FLT3 mutations.

## 2 MATERIALS AND METHODS

### 2.1 Selection procedures of eligible studies

The present meta‐analysis was conducted according to the Preferred Reporting Items for Systematic Reviews and Meta‐Analysis (PRISMA) statement^27^ and Meta‐Analysis of Observational Studies in Epiedemiology (MOOSE) guidelines^28^ (Supplementary PRISMA checklist and MOOSE checklist). In addition, PICOS framework was applied: population, AML patients with FLT3 mutations; intervention, flt3 inhibitors after allogeneic stem cell transplantation; comparison, no FLT3i maintenance or placebo; outcome, survival and relapse; study design, randomized controlled trials, retrospective and prospective comparative studies.

Systematic literature search was performed in PubMed, EMBASE, Web of Science, Cochrane Library and Clinicaltrials.gov from inception of each database to December 31, 2021. The following keywords were used for search: (TKI OR “tyrosine kinase inhibitor” OR FLT3 OR “fms‐like tyrosine kinase” OR sorafenib OR lestaurtinib OR midostaurin OR quizartinib OR gilteritinib OR crenolanib) AND (AML OR “acute myeloid leukemia”) AND (HSCT OR “stem cell transplant”). There was no language restriction. The reference lists of included articles were reviewed for additional eligible studies.

Two independent researchers initially screened the titles and abstracts for eligibility, and then reviewed the full texts for the final decision of included studies. Conflicts were resolved by further discussion with a third researcher. Included studies had met the following criteria: included FLT3‐mutated AML patients who received allo‐HSCT; used FLT3i for maintenance therapy after allo‐HSCT; was a retrospective or prospective study or randomized controlled trial (RCT) with a control comparison; and reported clinical outcomes regarding relapse or survival. Reviews, case series, basic researches and studies with duplicated datasets or providing incomplete date were excluded.

### 2.2 Endpoints and data extraction

The endpoints included overall survival (OS), relapse‐free survival (RFS), cumulative incidence of relapse (CIR), non‐relapse mortality (NRM), graft‐versus‐host disease (GVHD) and safety. Specifically, the safety was evaluated by grade ≥3 adverse events (AEs) and only RCTs were included as these trials documented AEs more accurately.

The following information were extracted from each study: first author, year of publication, study design, FLT3i regimens, comparators, sample size, age, gender and follow‐up duration. Furthermore, the characteristics of FLT3i administration and HSCT were extracted, which included median days of FLT3i starting after HSCT and median days of FLT3i use in the FLT3i group, and complete remission (CR) at transplant, cytogenetic risk, conditioning regimen, minimal residual disease (MRD) status at transplant, donor type and nucleophosmin 1 (NPM) mutation in both groups. Finally, we extracted hazard ration (HR) estimates and corresponding 95% confidence intervals (95%CIs) of CIR, OS, RFS, and events of NRM, GVHD and AEs in each group. If the study reported survival curves without HR estimates, we extracted the survival data from curves using Engauge Digitizer software and calculated the HR estimates and 95%CI.

### 2.3 Quality assessment and risk of bias

The quality of non‐RCT studies were assessed by using Newcastle‐Ottawa Scale (NOS), which assigned 9 stars to 8 items in 3 main domains. Studies with 5 and 6 stars were considered to have moderate quality and those with ≥7 stars were of high‐quality. The risk of bias of RCTs were judged according to Cochrane Collaboration's tool for assessing risk of bias. The risk of selection, performance, detection, attrition and reporting bias were classified as low, high or unknown levels.

Data extraction and assessment of study quality and risk of bias were also performed by two independent authors. Discrepancies were resolved by discussion with a third author.

### 2.4 Statistical analysis

The between‐study heterogeneity was assessed by *I* ^2^ statistic. The meta‐analysis was classified as having low, moderate and high heterogeneity if the *I* ^2^ was <25%, 25 ~ 50% and > 50%. A fixed‐effect model was used for data synthesis of low‐ and moderate‐heterogeneity analysis and a random‐effect model was applied if there was high heterogeneity. For OS, RFS and CIR outcomes, the pooled effect size was evaluated by HR estimates and corresponding 95%CI. For NRM, GVHD and AEs, the effect size was calculated using risk ratio (RR) estimates and 95%CI. Subgroups were divided according to FLT3i regimen (sorafenib, midostaurin, various FLT3i), study design (RCT, non‐RCT), and HR analysis model (univariate, multivariate). The publication bias was assessed by viewing the symmetry of funnel plot and Egger's test. If there was potential publication bias, a sensitivity analysis by trim‐and‐fill mothed, which conservatively imputed hypothetical negative unpublished studies to mirror the included positive studies that caused the asymmetry, was performed to judge whether the publication bias significantly influenced the pooled effect size. All quantitative analyses were performed by using STATA 16.0 (StataCorp, TX, USA). *p* < 0.05 was considered as statistical significance.

## 3 RESULTS

### 3.1 Description of eligible studies

**FIGURE 1:**
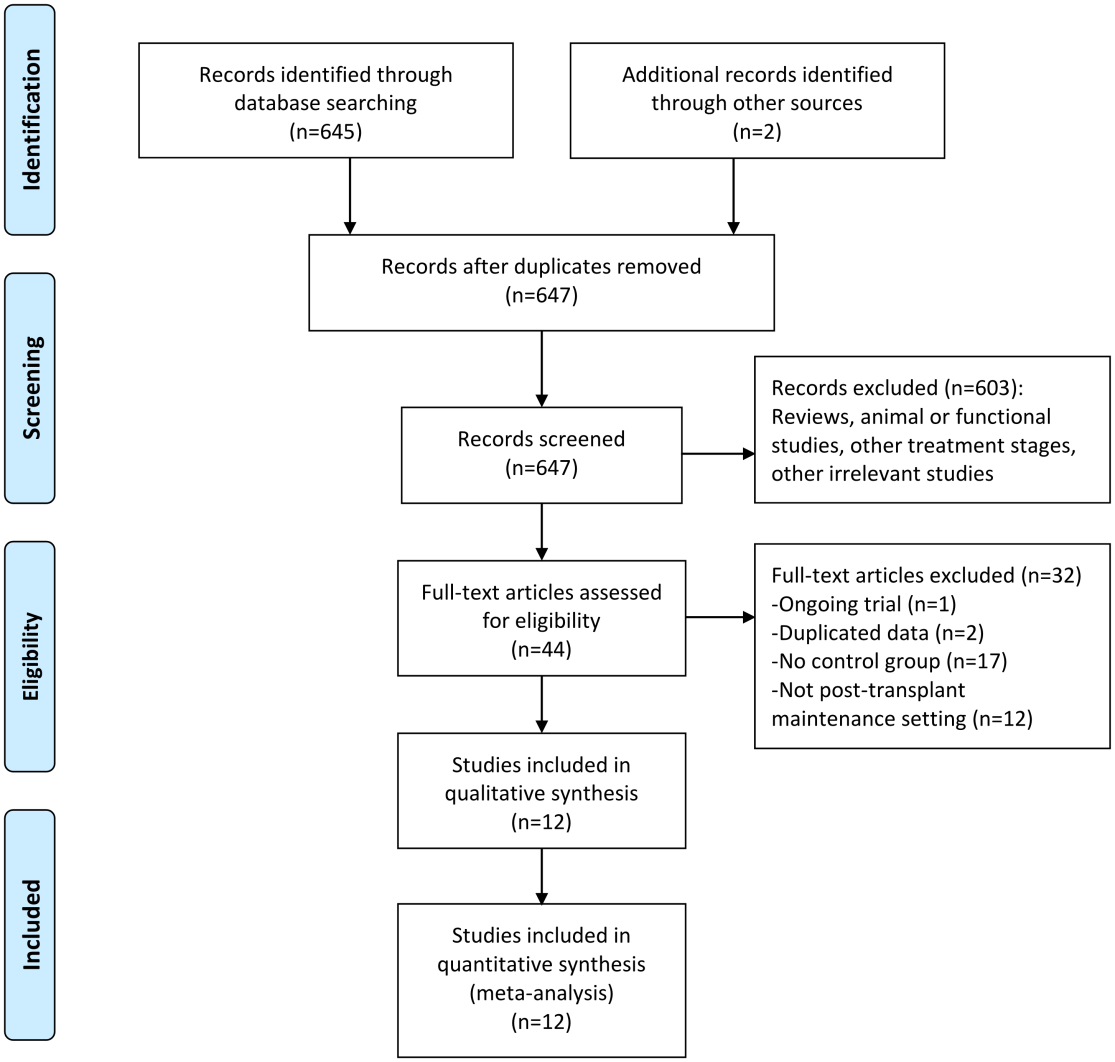
Flowchart of literature search and selection.

As shown in Figure 1, 647 articles were identified by literature search and 44 full‐text articles were further reviewed. Finally, 12 studies meeting the inclusion and exclusion criteria were remained for our meta‐analysis.^24^, ^25^, ^26^, ^29^, ^30^, ^31^, ^32^, ^33^, ^34^, ^35^, ^36^, ^37^ A total of 2282 FLT3‐mutated AML patients who had received HSCT were included, of which 635 cases were treated with FLT3i maintenance therapy and 1647 cases without FLT3i served as controls. Eight studies were of retrospective design.^26^, ^29^, ^31^, ^32^, ^33^, ^35^, ^36^, ^37^ One study was a prospective phase II trial and performed a landmark analysis in patients receiving allo‐HSCT without randomization.^34^ The other 3 were phase II or III RCTs that randomly assigned patients into FLT3i group or control group.^24^, ^25^, ^30^ Specifically, one study was a large‐sample‐size retrospective real‐world study that collected data of real‐world maintenance therapy after HSCT from multiple countries.^29^ Two studies were meeting abstracts^36^, ^37^ and the others were full‐text research articles.

Sorafenib and midostaurin were used in 9 and 2 studies, respectively. The real‐world study reported the usage of various FLT3i, including sorafenib, midostaurin, gilteritinib and quizartinib. All studies, except two, recruited FLT3‐ITD positive patients, of which a proportion had concomitant FLT3‐TKD mutations. Some patients only had FLT3‐TKD mutations, accounting for 2% in Bazarbachi's study and nearly 28% in Griffin’ study.^29^, ^32^ In 5 studies, all participants were in CR at the time of transplant,^24^, ^26^, ^30^, ^34^, ^37^ while in the other studies, only a proportion achieved CR before transplant. The features regarding cytogenetic risk, conditioning regimen, MRD status at transplant, donor type and concurrent NPM mutation were either not reported or distinct between different studies. All studies reported OS outcome, 10 RFS, 5 CIR, 6 aGVHD, 7 cGVHD and 6 NRM. The baseline characteristics of included studies were summarized in Table 1 and Table S1.

**TABLE 1:** Characteristics of studies included in meta‐analysis

Study	Design	FLT3i group				Control group				Median follow‐up, months	Outcome
		Regimen	N	Age, years	Sex	Comparator	N	Age, years	Sex		
Brunner (2016)	Retrospective	Sorafenib	26	55 (20–74)	12/14	No FLT3i	55	50 (25–73)	19/36	27.2	OS, RFS, CIR, NRM, GVHD
Ahmed (2017)	Retrospective	Sorafenib	12	NR	NR	No FLT3i	26	NR	NR	NR	OS, RFS
Xuan (2018)	Retrospective	Sorafenib	32	37 (15–55)	21/11	No FLT3i	50	34 (14–57)	28/22	23.6	OS, RFS, CIR, NRM, GVHD
Schlenk (2018)	Phase II trial	Midostaurin	71	NR	NR	No FLT3i	45	NR	NR	NR	OS, RFS
Bazarbachi (2019)	Retrospective	Sorafenib	28	50 (19–75)^a^	234/228^a^	No FLT3i	434			39.4	OS, RFS, CIR, NRM
Chappell (2019)	Retrospective	Sorafenib	29	49 (13–71)	12/17	No FLT3i	55	54 (10–71)	32/23	NR	OS, CIR, GVHD
Burchert (2020)	Phase II RCT	Sorafenib	43	54.2 (23.6–74.6)	18/25	Placebo	40	53.4 (18.6–75.6)	23/17	41.8	OS, RFS, NRM, GVHD, AE
Shi (2020)	Retrospective	Sorafenib	24	37 (14–62)	13/11	No FLT3i	32	39 (10–60)	17/15	NR	OS, RFS, NRM, GVHD
Xuan (2020)	Phase III RCT	Sorafenib	100	35 (26–42)	55/50	No FLT3i	102	35 (26–43)	52/50	21	OS, RFS, CIR, NRM, GVHD, AE
Maziarz (2020)	Phase II RCT	Midostaurin + SOC	30	48 (20–61)	16/14	SOC	30	56 (20–68)	18/12	24	OS, RFS, GVHD, AE
Morin (2020)	Retrospective	Sorafenib	20	NR	NR	No FLT3i	13	NR	NR	NR	OS
Griffin (2021)	Retrospective, real‐world	Various FLT3i	219	55.1 ± 13.5	145/74	No FLT3i	765	53.3 ± 12.9	460/35	NR	OS, RFS

*Note*: Age was presented as median and range or mean ± standard deviation. Sex was presented as the number of males and females.

Abbreviations: AE, adverse event; CIR, cumulative incidence of relapse; FLT3i, fms‐like tyrosine kinase 3 inhibitors; GVHD, graft‐versus‐host disease; NR, not reported; NRM, non‐relapse mortality; OS, overall survival; RCT, randomized controlled trial; RFS, relapse‐free survival; SOC, standard‐of‐care.

^a^ Median age and sex distribution of all participants.

According to NOS, 9 non‐RCTs had 6 to 9 stars and thus had moderate to high quality (Table S2). Among 3 RCTs, 2 were open‐label and 1 were double‐blind. There was low risk of attrition and reporting bias, and unknown risk of selection and detection bias. In overall, one RCT had low risk of bias and the other two had unknown or high risk of bias (Table S3).

### 3.2 OS

**FIGURE 2:**
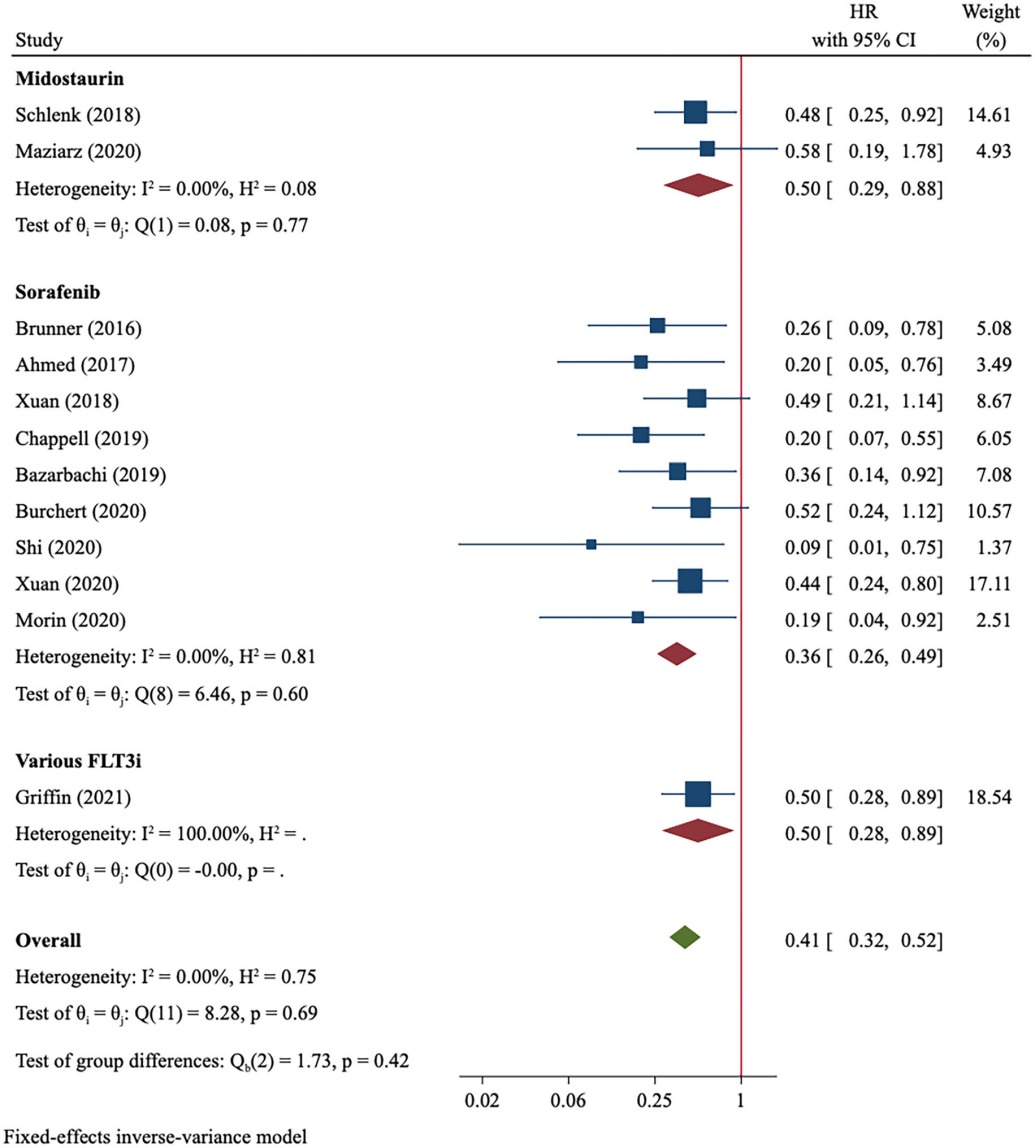
Overall survival of FLT3‐mutated AML patients treated with post‐transplant FLT3i maintenance compared to controls.

Twelve studies with 635 patients in FLT3i group and 1647 cases in control group were included in OS analysis. Among 10 studies with available data, 17.8% (69/388) of patients in FLT3i group and 40.0% (179/448) in control group died during follow‐ups (Table S4). There was no heterogeneity (*I* ^2^ = 0) and the fixed‐effect model was used for data synthesis. Meta‐analysis showed that FLT3i as maintenance therapy significantly improved OS of FLT‐mutated AML patients (HR = 0.41, 95%CI: 0.32–0.52, *p* < 0.001; Figure 2). Moreover, it seemed that sorafenib administration yielded more favorable OS than did midostaurin (sorafenib: HR = 0.36, 95%CI 0.26–0.49, *p* < 0.001; midostaurin: HR = 0.50, 95%CI 0.29–0.88, *p* = 0.017). Similarly, subgroup analysis of RCT yielded a more conservative association than that of non‐RCT (RCT: HR = 0.48, 95%CI 0.31–0.75; non‐RCT: HR = 0.37, 95%CI 0.28–0.50; Table S5). The exclusion of the large‐scale real‐world study did not significantly change the result (HR = 0.39, 95%CI 0.29–0.51).

### 3.3 RFS

**FIGURE 3:**
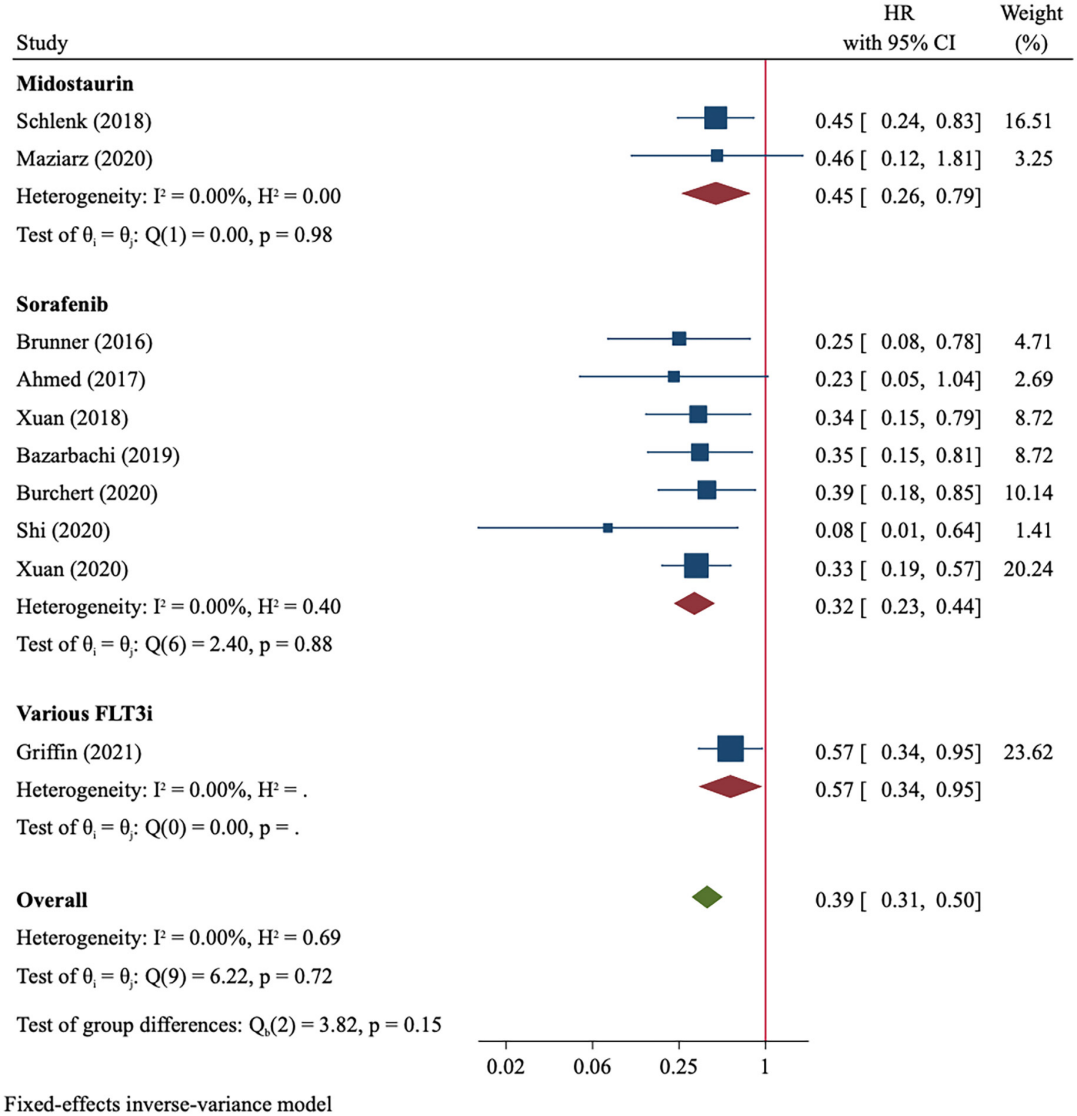
Relapse‐free survival of FLT3‐mutated AML patients treated with post‐transplant FLT3i maintenance compared to controls.

RFS outcome were compared between FLT3i and control groups in 10 studies that included 586 FLT3i‐treated patients and 1579 controls. The events of relapse and death occurred in 20.6% (70/339) of FLT3i‐treated patients and 46.6% (177/380) of controls (Table S4). Meta‐analysis using a fixed‐effect model demonstrated an improved RFS in favor of FLT3i (HR = 0.39, 95%CI 0.31–0.50, *p* < 0.001, *I* ^2^ = 0; Figure 3). Similar to what was observed in OS analysis, sorafenib might confer more RFS improvement than did midostaurin (sorafenib: HR = 0.32, 95%CI 0.23–0.44, *p* < 0.001; midostaurin: HR = 0.45, 95%CI 0.26–0.79, *p* = 0.005). The results did not differ between RCT and non‐RCT subgroups and was not significantly affected by the exclusion of the real‐word study (Table S5).

### 3.4 CIR

The CIR analysis included 5 eligible studies comprising 215 patients in FLT3i group and 696 patients in control group. A fixed‐effect model was used for data synthesis, demonstrating that FLT3i‐treated patients had a significantly lower CIR than controls (HR = 0.31, 95%CI 0.20–0.46, *p* < 0.001, *I* ^2^ = 0; Figure S1).

### 3.5 NRM

Of 6 eligible studies, 7.6% (19/251) and 11.6% (34/293) of patients in FLT3i and control groups, respectively, died from any cause not subsequent to relapse. However, meta‐analysis showed no significant NRM difference between both groups (RR = 0.69, 95%CI 0.41–1.17, *p* = 0.169; Figure S2).

### 3.6 GVHD

Any grade of aGVHD occurred in 92 out of 254 patients who received FLT3i for maintenance therapy and in 94 of 296 controls. The incidences of grade II‐IV aGVHD were 25.5% and 25.1% in FLT3i and control groups, respectively. The risk of aGVHD was not significant between both groups (overall aGVHD: RR = 1.17, 95%CI 0.93–1.47, *p* = 0.175; grade II‐IV aGVHD: RR = 1.03, 95%CI 0.74–1.45, *p* = 0.847; Figure S3). Similarly, the incidence of any grade of cGVHD and severe cGVHD did not differ between both groups (overall cGVHD: RR = 1.31, 95%CI 0.91–1.39, *p* = 0.276; severe cGVHD: RR = 1.39, 95%CI 0.62–3.13, *p* = 0.429; Figure S4).

### 3.7 Safety

**FIGURE 4:**
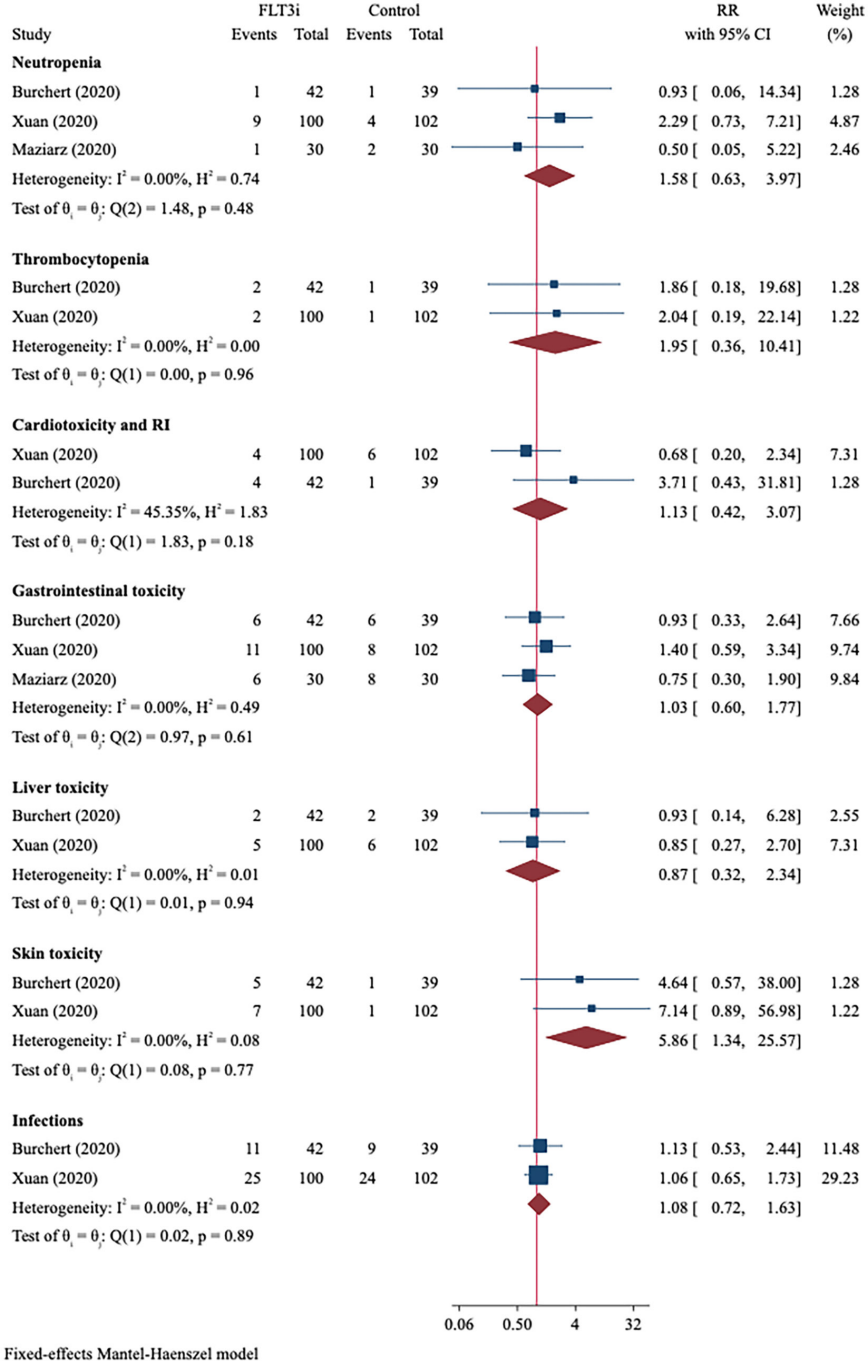
Grade ≥3 adverse events of FLT3‐mutated AML patients treated with post‐transplant FLT3i maintenance compared to controls.

As shown in Figure 4, the safety profiles of FLT3i group in terms of grade ≥3 cardiotoxicity and renal insufficiency, gastrointestinal toxicity, infections, liver toxicity, neutropenia, and thrombocytopenia were comparable to those of control group. However, the FLT3i‐treated patients had a higher risk of skin toxicity than controls (RR = 5.86, 95%CI 1.34–25.57, *p* = 0.019, Figure 4).

### 3.8 Publication bias

Publication bias was assessed in OS and RFS analyses as they had 10 or more eligible studies. The funnel plots were not symmetrical, and Egger's test indicated evidence of publication bias (*p* = 0.025 and 0.078, respectively). Then, we conducted a sensitivity analysis by trim‐and‐fill method. However, each inclusion of 3 imputed studies did not significantly affect the pooled effect size of OS (HR = 0.44, 95%CI 0.34–0.55, *p* < 0.001, Figure S5) and RFS (HR = 0.42, 95%CI 0.33–0.53, *p* < 0.001, Figure S6).

## 4 DISCUSSION

FLT mutation, which confers a worse prognosis, is a well‐characterized genetic abnormality for AML diagnosis, risk classification and management.^38^ Previous studies have demonstrated considerable efficacy and safety of FLT3 inhibitors in various settings of FLT3‐mutated AML patients.^39^ The addition of midostaurin to chemotherapy significantly prolonged OS than standard chemotherapy alone and was approved as first‐line treatment for FLT3‐mutated AML.^40^, ^41^ Despite not being approved for AML, the off‐label use of sorafenib has demonstrated potentials in improving clinical outcomes and survival in FLT3‐mutated AML patients.^42^ According to the newly released ELN Guideline for AML, FLT3 inhibitors have been recommended for intensive chemotherapy at induction, consolidation, maintenance and salvage stages of FLT3‐mutated patients.^38^

Given the high incidence of relapse post‐HSCT in FLT3‐mutated AML patients, post‐transplant maintenance therapy is urgently needed. In recent years, these FLT3 inhibitors have been explored for clinical value in preventing relapse and improving survival in this setting. Our meta‐analysis, incorporating current evidence from observational studies and randomized trials, demonstrated that FLT3i significantly prolonged OS and RFS and that sorafenib (no data for midostaurin) reduced relapse rate in the post‐transplant maintenance setting.

Despite no between‐study heterogeneity of the present meta‐analysis, some important influential factors on prognosis of FLT3‐mutated patients or HSCT‐treated patients were distinct among these studies. Concomitant NPM1 mutation was associated with lower relapse risk and longer OS, and pre‐transplant MRD status is a strong indicator of post‐HSCT relapse.^14^ Whether these factors can help to classify FLT3‐mutated patients who will gain benefit from post‐transplant FTL3i maintenance is not clear. In SORMAIN trial, sorafenib maintenance post‐HSCT conferred significant RFS benefit than placebo among the subgroups of FLT‐ITD^+^ patients who had a concomitant NPM1 mutation, undetectable MRD before HSCT or detectable MRD post‐HSCT.^25^ Patients without these features did not significantly benefit from sorafenib.^25^ However, the SORMAIN trial had a small sample size. A larger phase 3 trial conducted in China showed consistent relapse benefits regardless of the presence of NPM1 mutations and MRD status.^24^ More large‐scale, prospective, well‐designed trials are needed to validate the prognostic value of these factors.

Both midostaurin and sorafenib belong to the first‐generation FLT3 inhibitors that lack specificity for FLT3.^43^ Midostaurin has inhibitory activity against multiple receptor tyrosine kinases (RTKs), including FLT3, VEGFR, PDGFR, PKCα, c‐KIT et al. Sorafenib is another multi‐kinase inhibitor showing activity against FLT3, VEGFR and PDGFR kinases et al and has been approved for renal and liver cancers.^44^

First‐generation FLT3i may have some drug‐specific off‐target toxicities duo to the broad‐spectrum of kinase targets.^45^ Cardiovascular effects, such as cardiac failure, ischemia and QT prolongation, may be consequent to the inhibition of VEGFR,^46^ and myelosuppression may be linked to c‐KIT suppression.^47^ However, AEs are generally mild, and rates of grade ≥3 AEs are similar between FLT3i and control groups, except for skin toxicity, as indicated by our meta‐analysis of RCTs. Our meta‐analysis showed that sorafenib‐treated patients had higher risk of skin toxicity than controls. This off‐target skin reaction is inferred to be caused by the direct toxicity of sorafenib, which may be secreted into the eccrine glands, to skin.^48^ Despite the similar safety profiles, FLT3i group seemed to have a slightly higher rate of AE‐related discontinuations than control group.^25^, ^30^ Given the off‐target toxicities, some risk factors such as aneurysm or hypertension need to be considered before the first‐generation FLT3i usage, especially in real‐world setting, and the more selective second‐generation TKIs with fewer off‐target toxicities are in expectation.^45^

The present meta‐analysis supports the clinical application of the first‐generation FLT3i, mainly midostaurin and sorafenib, in the post‐transplant maintenance setting, but lacks evidence for the more specific, potent, second‐generation TKIs, such as crenolanib, quizartinib, gilteritinib.^45^ A real‐world study included 23 patients treated with gilteritinib or quizartinib but did not separately report their survival outcomes.^29^ Gilteritinib monotherapy led to longer OS, higher rate of complete remission, and fewer serious adverse events than salvage chemotherapy and was approved for relapsed or refractory FLT3‐mutated AML who have a very poor prognosis.^49^, ^50^ Another second‐generation TKI, quizartinib, also conferred survival benefits to this group of patients than salvage chemotherapy.^51^ Thus, the potential clinical benefit of these second‐generation TKIs as post‐transplant maintenance therapy is expected. A registered, large‐scale, phase 3 clinical trial (NCT02997202) for gilteritinib maintenance has been launched.

A recent meta‐analysis, by incorporating 7 eligible studies, has drawn similar conclusions that FLT3i maintenance reduces risk of relapse and death in FLT3‐mutated AML patients.^52^ Yet, the present meta‐analysis has several differences from the previous one. Our analysis includes more eligible studies and has a sample size three time as much as the previous one (2283 vs. 680), which provides a higher statistical power. Besides, the present analysis mainly uses HR estimate, a most appropriate statistic for time‐to‐event outcomes, while the previous one has only calculated RR estimates neglecting time‐to‐event information. Nonetheless, both meta‐analyses have demonstrated a significantly improved relapse and survival outcomes of FLT3‐mutated AML patients receiving FLT3i maintenance.

Some limitations of the present meta‐analysis should be noted. Firstly, AML patients had highly heterogeneous clinical characteristics in terms of pre‐transplant TKIs usage, conditioning intensity, pre‐ and post‐transplant MRD status, FLT3i administration schedule, and NPM1 co‐mutations. This may limit the interpretation of our meta‐analysis in different patient subgroups and patient‐level data need to be collected for a precise assessment. Secondly, most of included studies are retrospective and observational with low evidence level, and only 3 prospective RCTs were available. Thirdly, the sample size is still small. Eight studies included less than 100 patients and the phase 2 RAIUS trial had too small sample size to reach statistical significance.^30^ Thus, more large‐scale, randomized trials are needed to validate the efficacy and safety of FLT3i as post‐transplant maintenance therapy.

## 5 CONCLUSION

In summary, the present meta‐analysis demonstrates that FLT3i maintenance therapy following allo‐HSCT, mainly midostaurin and sorafenib, can reduce relapse risk and prolong survival in FLT3‐mutated AML patients, and that the inhibitors are well tolerated.

## AUTHOR CONTRIBUTIONS

**Xinhong Fei:** Conceptualization (equal); formal analysis (lead); writing – original draft (lead); writing – review and editing (equal). **Shuqin Zhang:** Data curation (equal); formal analysis (supporting); writing – original draft (supporting); writing – review and editing (equal). **Jiangying Gu:** Data curation (equal); formal analysis (supporting); writing – original draft (supporting); writing – review and editing (equal). **Jingbo Wang:** Conceptualization (equal); supervision (lead); writing – review and editing (equal).

## FUNDING INFORMATION

This work was supported by China Capital Characteristic Clinic Project (Grant No. Z211100002921037).

## CONFLICT OF INTEREST

The authors have no conflict of interest.

## ETHICS APPROVAL STATEMENT

Not applicable.

## PATIENT APPROVAL STATEMENT

Not applicable.

## Supporting information


Figure S1.

Figure S2.

Figure S3.

Figure S4.

Figure S5.

Figure S6.

Table S1.
Table S2.
Table S3.
Table S4.
Table S5.

## Data Availability

The data that support the findings of this study are available on request from the corresponding author.
