# Supplementary figures and images for: FLT3 inhibitors as maintenance therapy post allogeneic hematopoietic stem cell transplantation in acute myeloid leukemia patients with FLT3 mutations: A meta‐analysis
Source: Cancer Med. 2022 Nov 21;12(6):6877–88. doi: 10.1002/cam4.5480 (PMC10067110; doi:10.1002/cam4.5480)

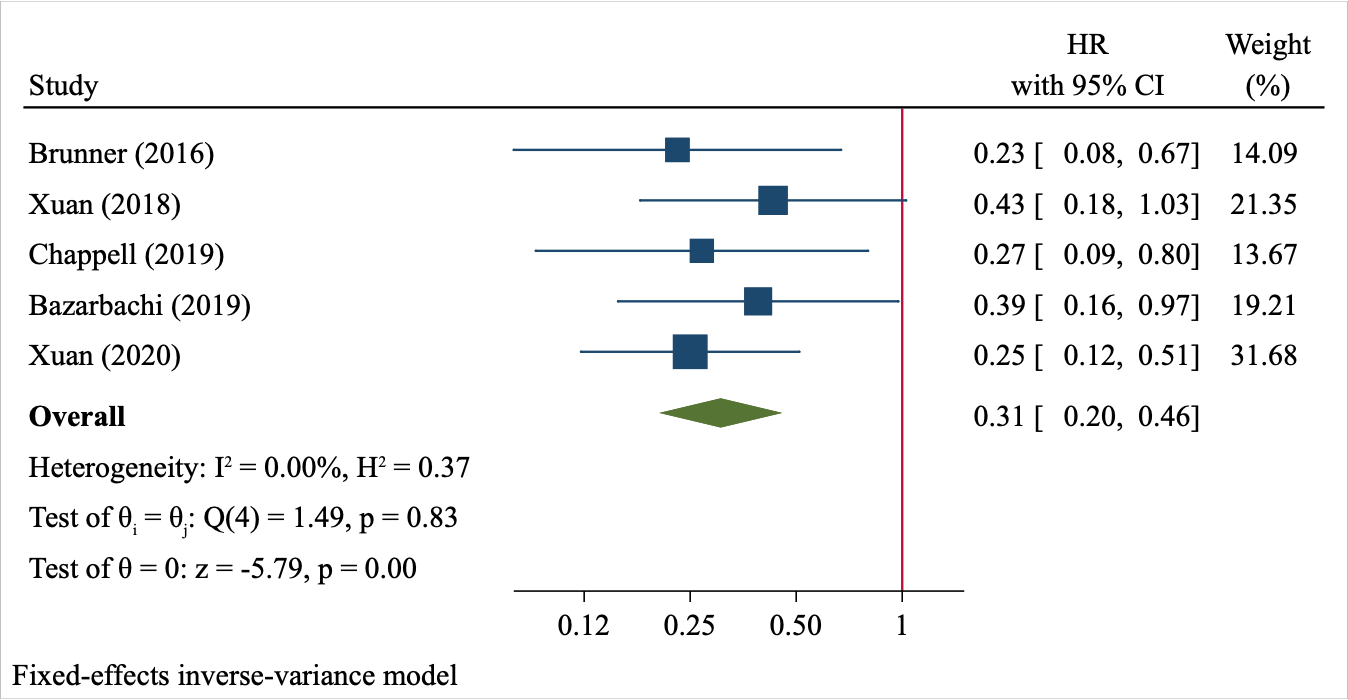

Supplement: Supplementary file 1 — Figure S1. [file CAM4-12-6877-s003.tif]

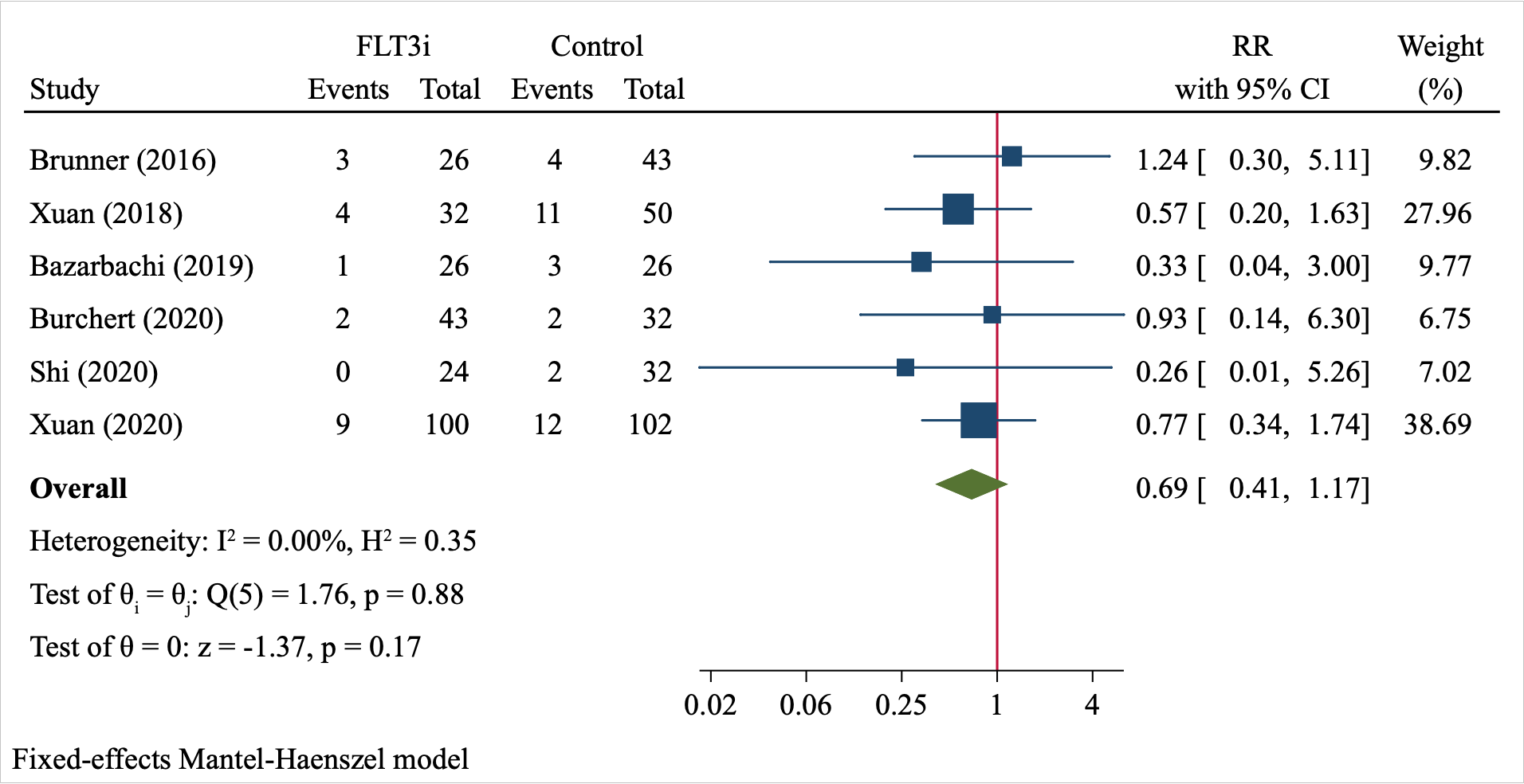

Supplement: Supplementary file 2 — Figure S2. [file CAM4-12-6877-s005.tif]

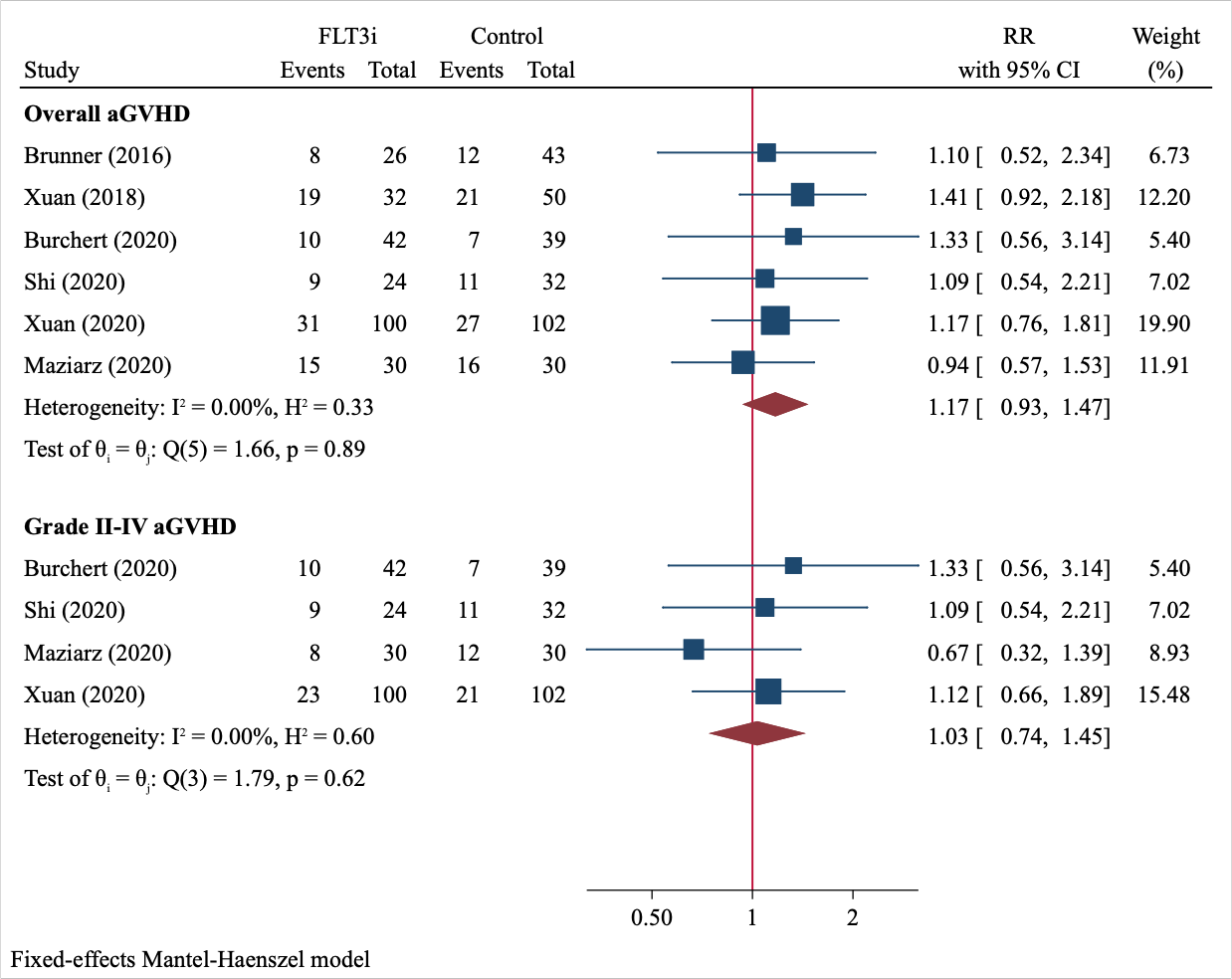

Supplement: Supplementary file 3 — Figure S3. [file CAM4-12-6877-s007.tif]

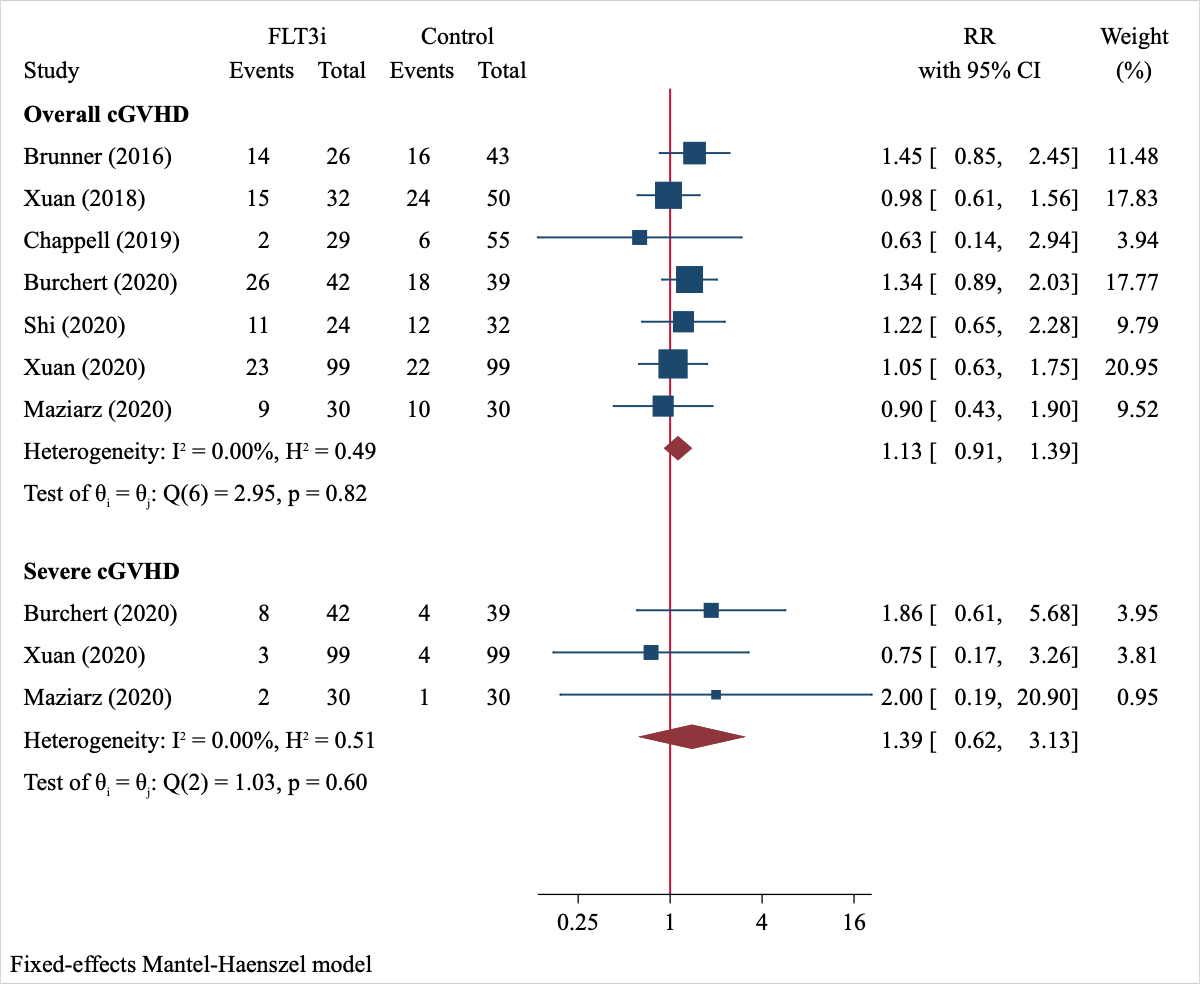

Supplement: Supplementary file 4 — Figure S4. [file CAM4-12-6877-s006.tif]

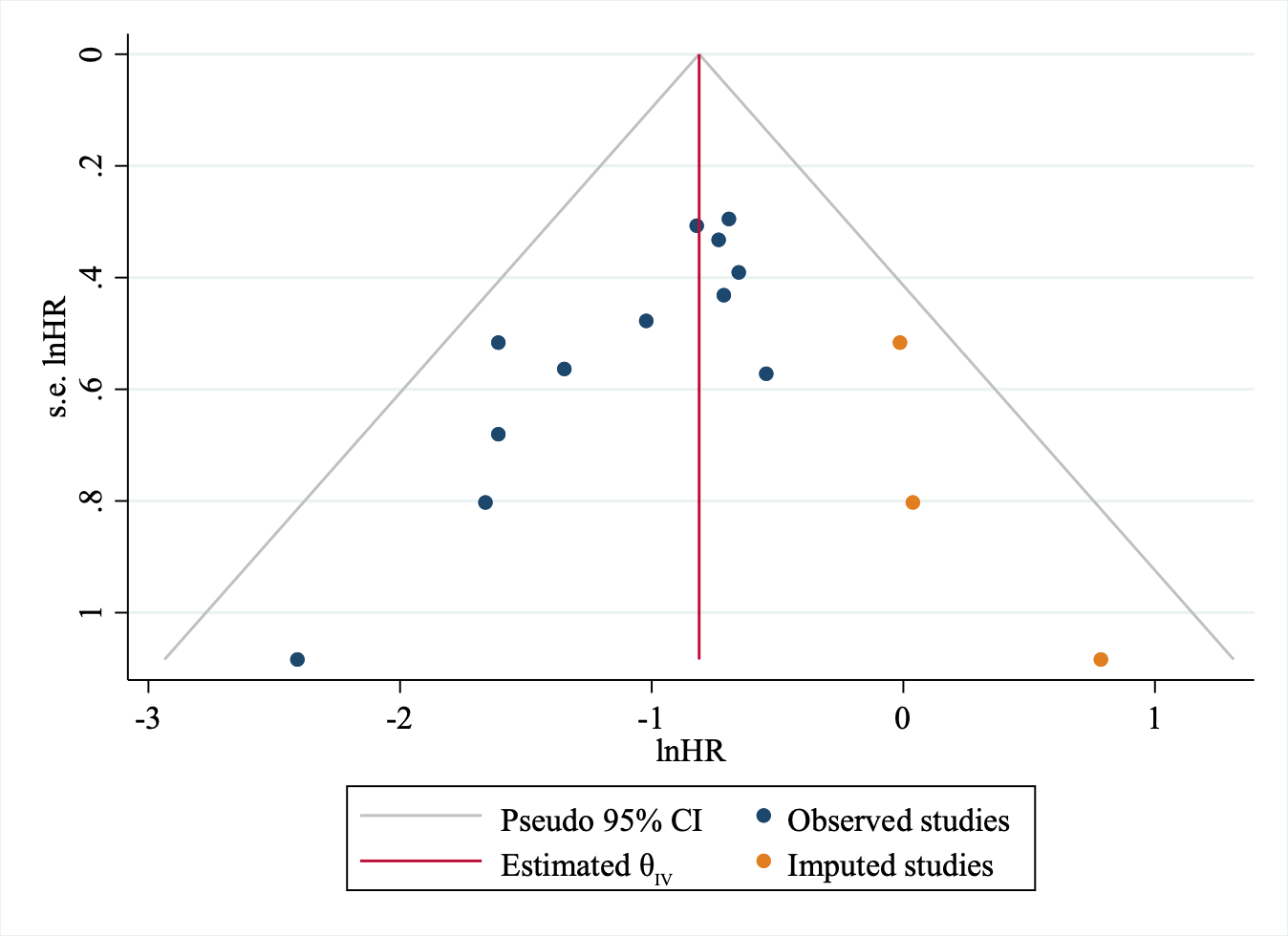

Supplement: Supplementary file 5 — Figure S5. [file CAM4-12-6877-s002.tif]

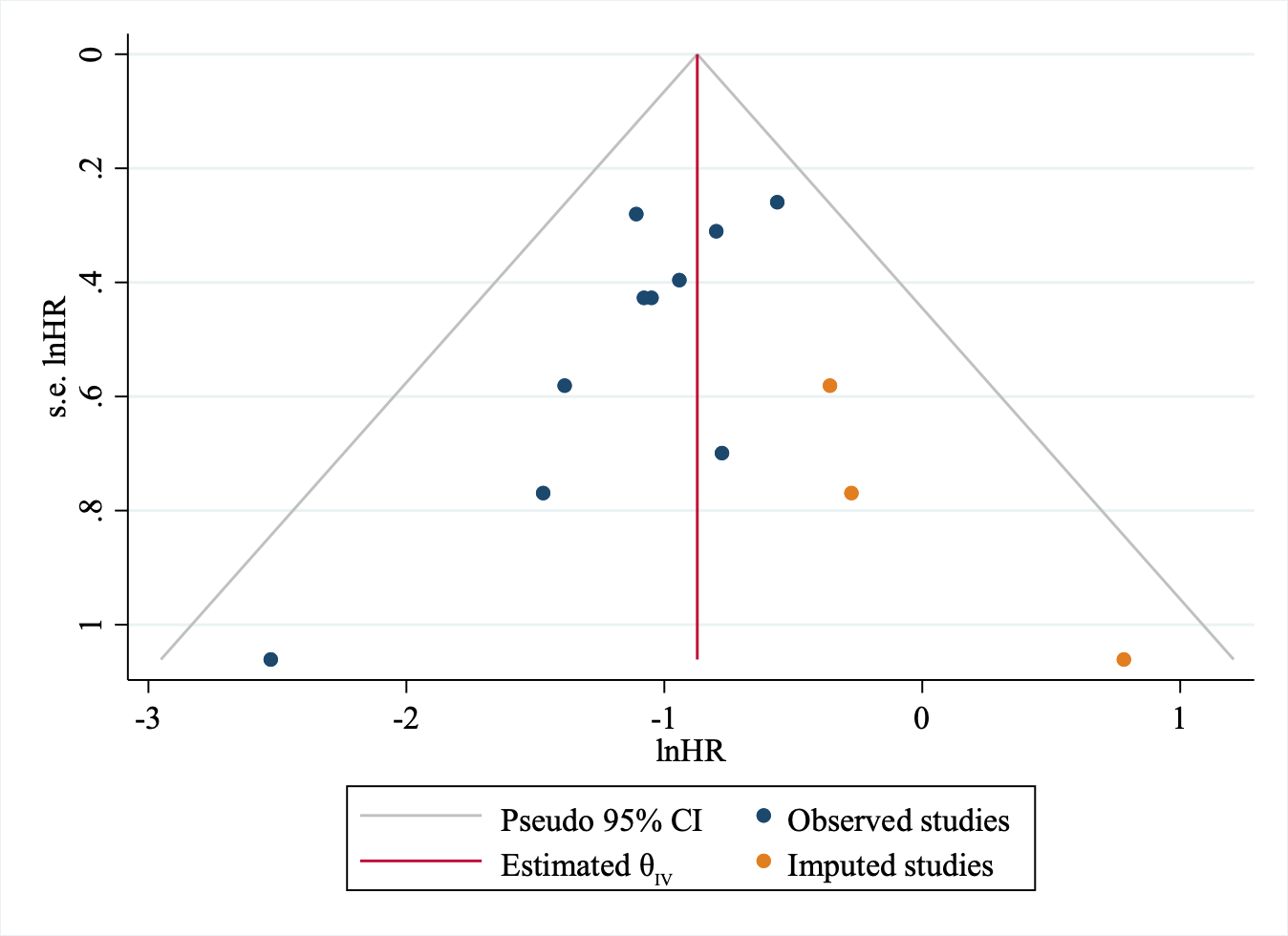

Supplement: Supplementary file 6 — Figure S6. [file CAM4-12-6877-s001.tif]
