# Supplementary material for: FLT3 inhibitors as maintenance therapy post allogeneic hematopoietic stem cell transplantation in acute myeloid leukemia patients with FLT3 mutations: A meta‐analysis
Source: Cancer Med. 2022 Nov 21;12(6):6877–88. doi: 10.1002/cam4.5480 (PMC10067110; doi:10.1002/cam4.5480)
Supplement: Supplementary file 7 — Table S1. Table S2. Table S3. Table S4. Table S5. [file CAM4-12-6877-s004.docx]

Table S1. FLT3i administration, disease status and transplant characteristics of studies included in meta-analysis.

| Study | Group | Sample size | CR status at transplant (Yes/No) | Median days of FLT3i starting after HSCT (range) | Median days of FLT3i use (range) | Cytogenetic risk^‡^ | Conditioning regimen (MAC/RIC) | MRD status at transplant^§^ | Donor type | NPM1 mutation^§^ |
| --- | --- | --- | --- | --- | --- | --- | --- | --- | --- | --- |
| Brunner, 2016 | Sorafenib | 26 | 100% CR1 | 68 (36-193) | NR | 1/23/2 | 14/12 | NR | 21 matched, 5 mismatched | 14/12 |
|  | Control | 55 | 100% CR1 |  |  | 2/47/4 | 27/28 | NR | 45 matched, 10 mismatched | 21/34 |
| Ahmed, 2017 | Sorafenib | 13 | 27/12^†^ | NR | NR | 0/9/30^#^ | 13/0 | NR | NR | NR |
|  | Control | 26 |  |  |  |  | 26/0 | NR | NR | NR |
| Xuan, 2018 | Sorafenib | 32 | 28/4 | 30 (30-131) | NR | 2/28/2 | 32/0 | NR | 17 MSD, 3 MUD, 12 haplo | NR |
|  | Control | 50 | 37/13 |  |  | 0/48/2 | 50/0 | NR | 21 MSD, 8 MUD, 21 haplo | NR |
| Schlenk, 2018 | Midostaurin | 71 | 100% CR1 | 71 (31-100) | NR | NR | NR | NR | NR | NR |
|  | Control | 45 | 100% CR1 |  |  | NR | NR | NR | NR | NR |
| Bazarbachi, 2019 | Sorafenib | 28 | 378/84^†^ | 55 (1-173) | NR | NR | NR | NR | NR | NR |
|  | Control | 434 |  |  |  | NR | NR | NR | NR | NR |
| Chappell, 2019 | Sorafenib | 29 | NR | 78 (36-297) | 381 (18-805) | NR | 29/0 | NR | 10 MSD, 14 MUD, 2 mMUD, 1 mMRD | NR |
|  | Control | 55 | NR |  |  | NR | 43/12 | NR | 21 MSD, 19MUD, 9 mMUD | NR |
| Burchert, 2020 | Sorafenib | 43 | 36/7 | NR | 242 (9-748) | 0/40/1 | 18/25 | 27/9 | 35 MUD, 8 MSD | 29/43 |
|  | Control | 40 | 31/9 |  |  | 0/36/3 | 19/21 | 19/12 | 28 MUD, 12 MSD | 23/40 |
| Shi, 2020 | Sorafenib | 24 | 19/5 | 83 (37-222) | 238 (21-385) | NR | NR | 12/12 | 7 matched, 17 mismatched | 8/16 |
|  | Control | 32 | 23/9 |  |  | NR | NR | 18/14 | 15 matched,17 mismatched | 10/22 |
| Xuan, 2020 | Sorafenib | 100 | 100/0 | 30 (30-42) | 134 (116-150) | 6/80/7 | NR | 31/69 | 44 MSD, 8 MUD, 48 haplo | 29/71 |
|  | Control | 102 | 102/0 |  |  | 4/85/5 | NR | 34/68 | 39 MSD, 6 MUD, 57 haplo | 26/76 |
| Maziarz, 2020 | Midostaurin | 30 | 30/0 | NR | NR | NR | 30/0 | NR | NR | NR |
|  | Control | 30 | 30/0 |  |  |  | 30/0 | NR | NR | NR |
| Morin, 2020 | Sorafenib | 20 | 20/0 | 68 (40-213) | 180 (1-765) | NR | NR | NR | NR | NR |
|  | Control | 13 | 13/0 |  |  | NR | NR | NR | NR | NR |
| Griffin, 2021 | Various FLT3i | 219 | 208/11 | NR | NR | 30/83/104 | NR | 84/111 | NR | NR |
|  | Control | 765 | 745/20 |  |  | 158/332/262 | NR | 203/434 | NR | NR |

† Data for all patients.

‡ Indicating favorable, intermediate and adverse risk.

§ Indicating positive and negative patients.

FLT3i: fms-like tyrosine kinase 3 inhibitors. CR: complete remission; CR1: first CR; HSCT: hematopoietic stem cell transplantation; MAC: myeloablative conditioning; RIC: reduced intensity conditioning; MRD: minimal residual disease. MSD: matched sibling donor; MUD: matched unrelated donor; mMUD: mismatched unrelated donor; mMRD: mismatched related donor; haplo: haplo-identical donor; NPM1: nucleophosmin 1; NR: not reported.

Table S2 Quality assessment of non-RCT studies included in meta-analysis according to Newcastle-Ottawa Scale.

| Study | Selection domain | | | | Comparability domain | Outcome domain | | | Total stars |
| --- | --- | --- | --- | --- | --- | --- | --- | --- | --- |
|  | Representativeness of the exposed cohort | Selection of the non-exposed cohort | Ascertainment of exposure | Demonstration that outcome of interest was not present at start of study | Comparability of cohorts on the basis of the design or analysis | Assessment of outcome | Was follow-up long enough for outcome to occur | Adequacy of follow-up of cohorts |  |
| Brunner, 2016 | * | * | * | * | ** | * | * | * | 9 |
| Ahmed, 2017 | * | * | * | * |  | * |  | * | 6 |
| Xuan, 2018 | * | * | * | * | ** | * | * | * | 9 |
| Schlenk, 2018 | * | * | * | * |  | * |  | * | 6 |
| Bazarbachi, 2019 | * | * | * | * |  | * | * | * | 7 |
| Chappell, 2019 | * | * | * | * |  | * |  | * | 6 |
| Shi, 2020 | * | * | * | * | * | * |  | * | 7 |
| Morin, 2020 | * | * | * | * |  | * |  | * | 6 |
| Griffin, 2021 | * | * | * | * | * | * |  | * | 7 |

Table S3. Quality assessment of RCTs according to Cochrane Collaboration’s tool for assessing risk of bias.

| Trial | Random sequence generation (selection bias) | Allocation concealment (selection bias) | Blinding of participants and personnel (performance bias) | Blinding of outcome assessment (detection bias) | Incomplete outcome data (attrition bias) | Selective reporting (reporting bias) |
| --- | --- | --- | --- | --- | --- | --- |
| Burchert, 2020 | Low | Unknown | Low | Unknown | Low | Low |
| Xuan, 2020 | Low | Unknown | High | Unknown | Low | Low |
| Maziarz, 2020 | Unknown | Unknown | High | Unknown | Low | Low |

Table S4. Detailed data of OS, RFS and CIR used in meta-analysis.

| Outcome | FLT3i (events/total) | Control (events/total) | HR (95%CI) | Analysis |
| --- | --- | --- | --- | --- |
| OS |  |  |  |  |
| Brunner, 2016 | 4/26 | 23/55 | 0.26 (0.09-0.82) | Multivariate |
| Ahmed, 2017 | 0/13 | 10/26 | 0.20 (0.05-0.72) ^†^ | Univariate |
| Xuan, 2018 | 7/32 | 23/50 | 0.49 (0.21-1.14) ^†^ | Univariate |
| Schlenk, 2018 | 20/71 | 18/45 | 0.48 (0.25-0.92) ^†^ | Univariate |
| Chappell, 2019 | 2/29 | 29/55 | 0.20 (0.07-0.53) | Univariate |
| Bazarbachi, 2019 | NA/28 | NA/434 | 0.36 (0.14-0.91) | Multivariate |
| Burchert, 2020 | 11/43 | 16/40 | 0.52 (0.24-1.11) | Univariate |
| Shi, 2020 | 1/24 | 12/32 | 0.09 (0.01-0.70) ^†^ | Univariate |
| Xuan, 2020 | 17/100 | 33/102 | 0.44 (0.24-0.80) | Multivariate |
| Maziarz, 2020 | 5/30 | 8/30 | 0.58 (0.19-1.79) | Univariate |
| Morin, 2020 | 2/20 | 7/13 | 0.19 (0.04-0.93) ^†^ | Univariate |
| Griffin, 2021 | NA/219 | NA/765 | 0.50 (0.28-0.89) | Multivariate |
| RFS |  |  |  |  |
| Brunner, 2016 | 5/26 | 26/55 | 0.25 (0.08-0.78) | Multivariate |
| Ahmed, 2017 | 2/13 | 14/26 | 0.23 (0.05-1.02) ^†^ | Univariate |
| Xuan, 2018 | 7/32 | 33/50 | 0.34 (0.15-0.80) | Multivariate |
| Schlenk, 2018 | 22/71 | 21/45 | 0.45 (0.24-0.81) ^†^ | Univariate |
| Bazarbachi, 2019 | NA/28 | NA/434 | 0.35 (0.15-0.80) | Multivariate |
| Burchert, 2020 | 10/43 | 19/40 | 0.39 (0.18-0.85) | Univariate |
| Shi, 2020 | 1/24 | 13/32 | 0.08 (0.01-0.64) ^†^ | Univariate |
| Xuan, 2020 | 20/100 | 44/102 | 0.33 (0.19-0.57) | Multivariate |
| Maziarz, 2020 | 3/30 | 7/30 | 0.46 (0.12-1.86) | Univariate |
| Griffin, 2021 | NA/219 | NA/765 | 0.57 (0.34-0.94) | Multivariate |
| CIR |  |  |  |  |
| Brunner, 2016 | 2/26 | 21/55 | 0.23 (0.08-0.68) | Univariate |
| Xuan, 2018 | 6/32 | 23/50 | 0.43 (0.16-0.91) | Multivariate |
| Chappell, 2019 | 3/29 | 30/55 | 0.27 (0.09-0.79) | Univariate |
| Bazarbachi, 2019 | NA/28 | NA/434 | 0.39 (0.16-1.00) | Multivariate |
| Xuan, 2020 | 20/100 | 44/102 | 0.25 (0.12-0.50) | Multivariate |

† Calculated by using survival data curated from survival curves.

FLT3i: fms-like tyrosine kinase 3 inhibitors; OS: overall survival; RFS: relapse-free survival; CIR: cumulative incidence of relapse; HR: hazard ratio; CI: confidence interval; NA: not available.

Table S5. Subgroup analyses of OS, RFS and CIR between FLT3i and control groups.

| Outcome | No. of studies | Sample size | I^2^ (%) | HR | 95%CI | P |
| --- | --- | --- | --- | --- | --- | --- |
| OS |  |  |  |  |  |  |
| RCT | 3 | 173/172 | 0 | 0.48 | 0.31-0.75 | 0.001 |
| Non-RCT | 9 | 462/1475 | 0 | 0.37 | 0.28-0.50 | <0.001 |
| Univariate analysis | 8 | 262/291 | 0 | 0.39 | 0.28-0.55 | <0.001 |
| Multivariate analysis | 4 | 373/1756 | 0 | 0.42 | 0.39-0.61 | <0.001 |
| Excluding real-world study^†^ | 11 | 416/882 | 0 | 0.39 | 0.29-0.51 | <0.001 |
| RFS |  |  |  |  |  |  |
| RCT | 3 | 173/172 | 0 | 0.36 | 0.23-0.55 | <0.001 |
| Non-RCT | 7 | 413/1407 | 0 | 0.41 | 0.30-0.56 | <0.001 |
| Univariate analysis | 5 | 181/173 | 0 | 0.38 | 0.25-0.58 | <0.001 |
| Multivariate analysis | 5 | 405/1406 | 0 | 0.40 | 0.29-0.54 | <0.001 |
| Excluding real-world study^†^ | 9 | 367/814 | 0 | 0.35 | 0.26-0.46 | <0.001 |
| CIR |  |  |  |  |  |  |
| RCT | 1 | 100/102 | - | 0.25 | 0.12-0.51 | <0.001 |
| Non-RCT | 4 | 115/594 | 0 | 0.34 | 0.21-0.55 | <0.001 |
| Univariate analysis | 2 | 55/110 | 0 | 0.25 | 0.12-0.53 | <0.001 |
| Multivariate analysis | 3 | 160/586 | 0 | 0.33 | 0.21-0.53 | <0.001 |

† The study of Griffin *et al*, 2021 (ref. 29).

FLT3i: fms-like tyrosine kinase 3 inhibitors; OS: overall survival; RFS: relapse-free survival; CIR: cumulative incidence of relapse; RCT: randomized controlled trial.
